# A Recurrent Case of Papillary Eccrine Adenoma

**DOI:** 10.7759/cureus.65539

**Published:** 2024-07-27

**Authors:** Renee Chang, Armaan Guraya, Renee Lucero, Paul Shitabata

**Affiliations:** 1 Dermatology, Touro College of Osteopathic Medicine, Henderson, USA; 2 Dermatology, Prime West Consortium, Newport Beach, USA; 3 Dermatopathology, Prime West Consortium, Newport Beach, USA

**Keywords:** recurrence, tubopapillary adenoma, adnexal carcinoma mimic, eccrine gland neoplasm, papillary eccrine adenoma

## Abstract

Papillary eccrine adenoma (PEA) is a rare benign eccrine gland neoplasm presenting as a solitary nodule, primarily in middle-aged African American females. Accurate histological diagnosis is crucial due to its potential to mimic adnexal carcinomas. Complete excision is recommended due to its risk of local aggression and recurrence. A 75-year-old Caucasian male with a history of basal cell carcinoma (BCC) presented with a recurrent pink, scaly nodule on the right medial pretibial leg area. Initial biopsy showed benign PEA. The lesion recurred after one year, and a re-biopsy confirmed a tubulopapillary adenoma within a scar. The lesion was excised with a 2 mm margin. PEA is characterized histologically by dilated ducts lined by a dual layer of tumor cells, often with intraluminal papillae structures. Immunohistochemical staining aids diagnosis, with markers such as S-100, carcinoembryonic antigen (CEA), and epithelial membrane antigen (EMA) indicating eccrine differentiation. Differential diagnoses include adnexal carcinomas and BCC with eccrine differentiation. Complete excision is necessary to prevent recurrence.

## Introduction

Papillary eccrine adenoma (PEA) is a rare benign eccrine gland neoplasm that typically arises in the distal extremities and appears as a solitary, firm, dome-shaped cutaneous nodule [1,2]. The nodule is normally small, ranging from 0.5 to 2 cm in diameter, and can vary in color, including red, brown, or gray [3]. This adenoma more commonly affects middle-aged African American females [2,3]. It is imperative that PEA is accurately diagnosed histologically as it can mimic adnexal carcinomas [4]. Although slow-growing and benign, complete excision of a PEA may be necessary as it can be locally aggressive and recurrent [5]. The objective of this case report is to improve recognition of this lesion and to better understand how to distinguish it from its malignant counterpart, adnexal carcinomas.

## Case presentation

**Figure 1:**
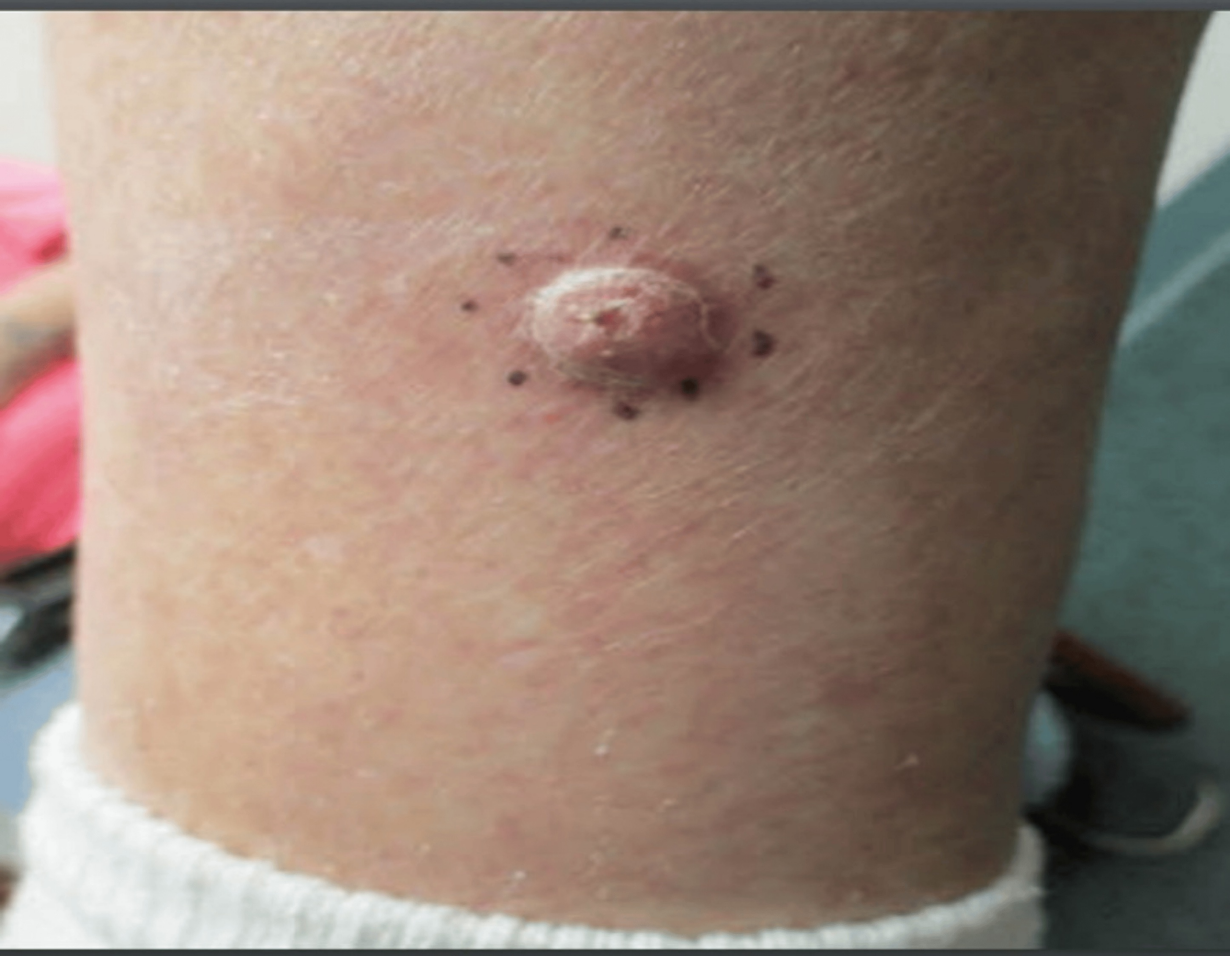
Right medial pretibial leg with a large pink, centrally eroded nodule

**Figure 2:**
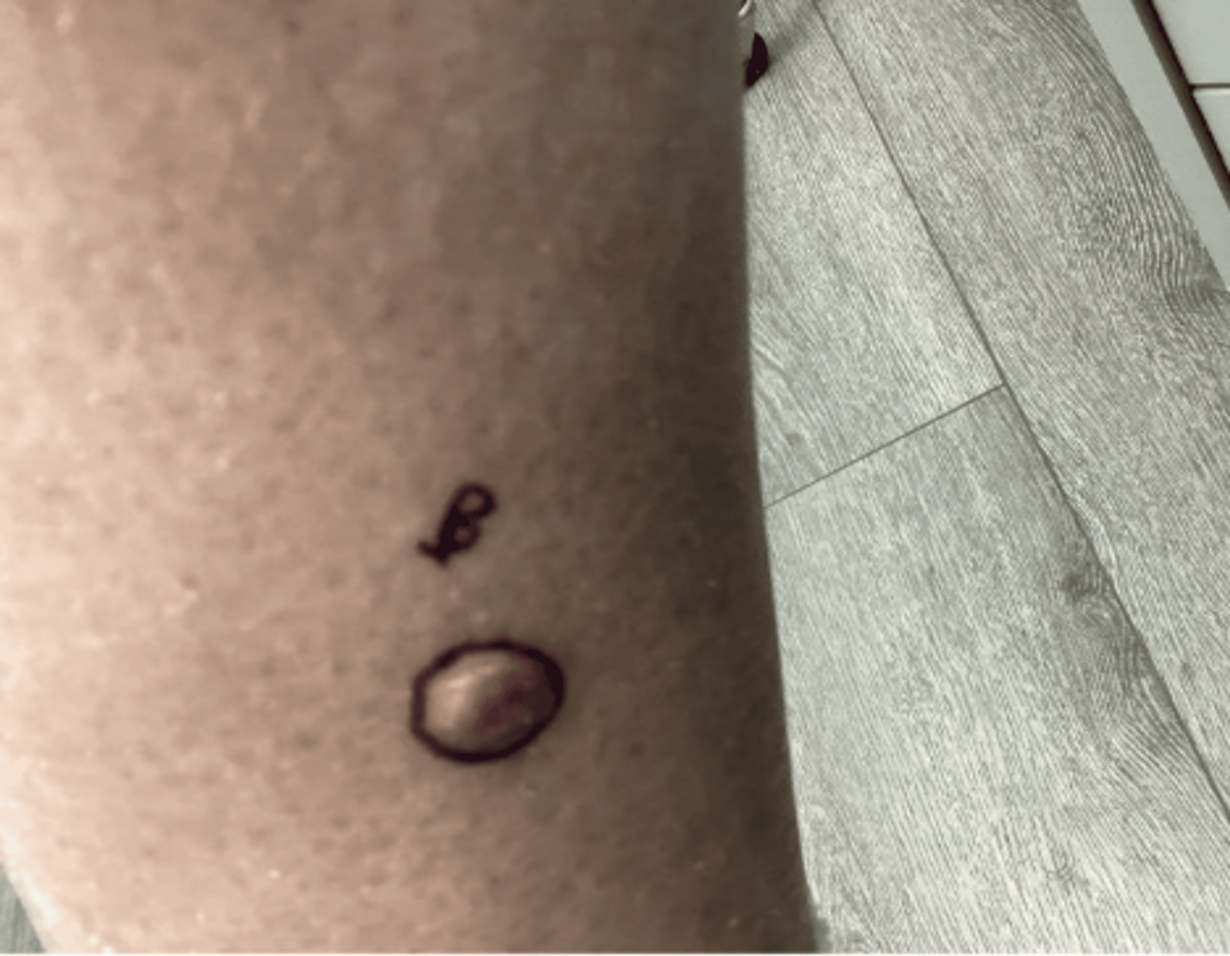
Right medial pretibial leg with recurrent smooth pink nodule

**Figure 3:**
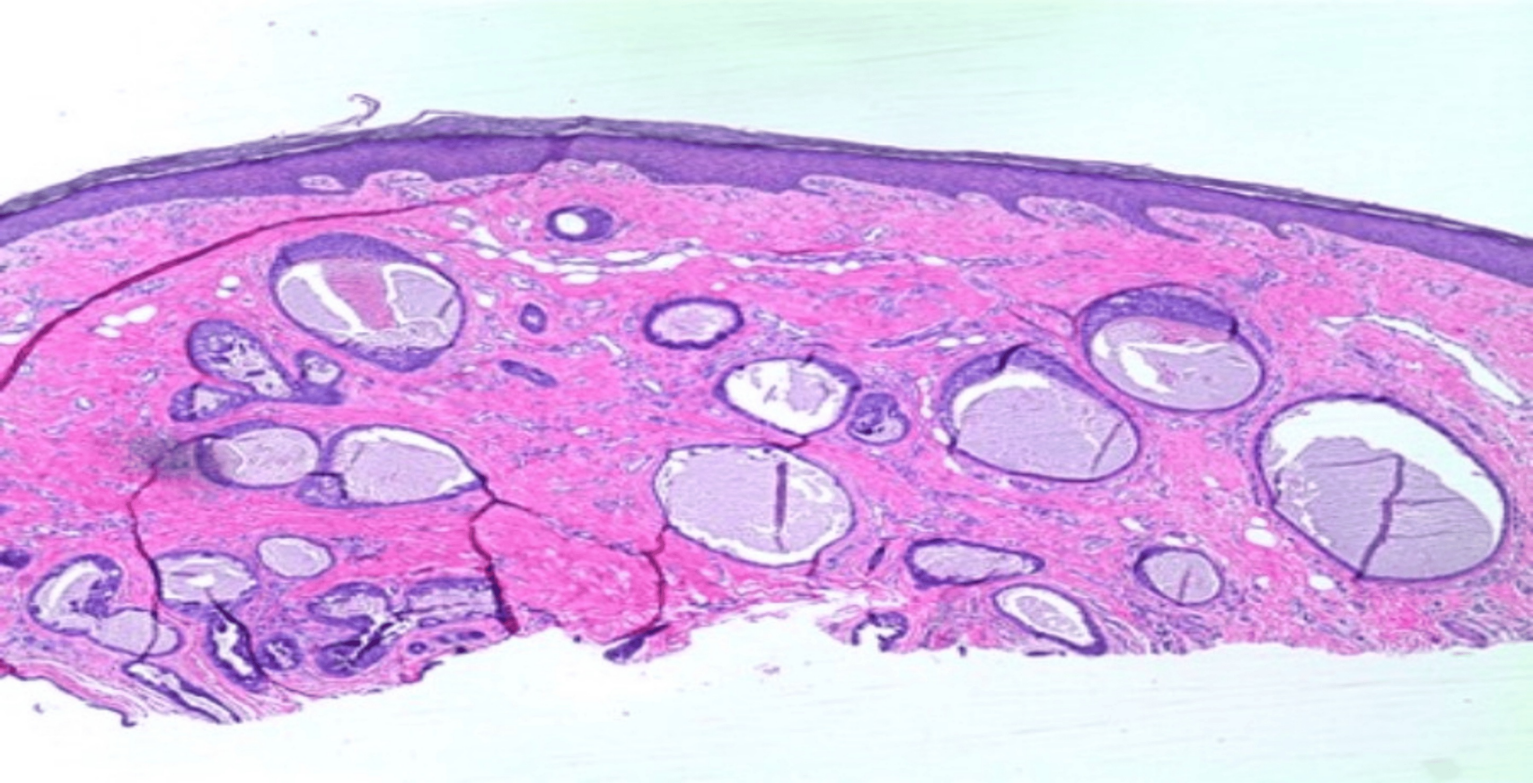
Biopsy showing a proliferation of numerous epithelial strands and tubules with lumens lined by cells of multilayered lining (4x magnification, hematoxylin and eosin staining)

**Figure 4:**
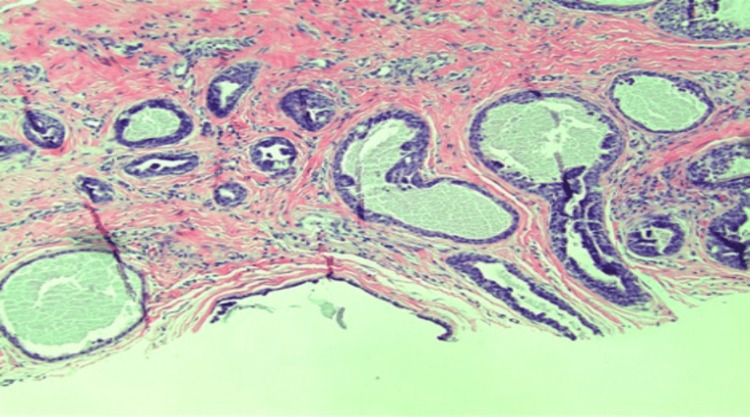
There is a proliferation of numerous epithelial strands and tubules with lumens lined by cells of multilayered lining. Focal papillary morphology and tiny areas of necrosis are noted (10x magnification, hematoxylin and eosin staining)

We present a case of a 75-year-old Caucasian male with a history of basal cell carcinoma (BCC). He first presented with a 1.1 cm, pink, scaly, and eroded nodule in the right medial pretibial leg that was present for years (Figure 1). A shave biopsy showed a benign PEA, extending to the deep margin. No evidence of malignancy was noted. The patient returned over one year later with a 1.2 cm smooth, round, pink nodule at the site of the previous biopsy (Figure 2). Re-biopsy of the lesion once again revealed a tubulopapillary adenoma within a scar (Figures 3-4). The sample demonstrated a syringoma-like example of tubular adenoma that persisted after the previous biopsy. The histopathological features in the second biopsy specimen did not suggest adnexal carcinoma. The lesion was then excised with a 2 mm margin due to recurrence and risk of local invasion. Final pathology of the excision showed a proliferation of numerous epithelial strands and tubules with lumens lined by cells of multilayered lining, consistent with PEA. 

## Discussion

PEA is a benign neoplasm of the eccrine glands with distinct histological features that can pose diagnostic challenges due to its rarity and ability to mimic adnexal carcinomas. Differential diagnoses include adnexal carcinomas and BCC with eccrine differentiation [6]. Histologically, PEA involves a nonmalignant growth of ducts within the sweat glands, located within the dermis. These ducts are often observed to be dilated, featuring a dual layer of tumor cells. The inner epithelial cell layer frequently exhibits characteristic intraluminal papillations in a cribiform growth pattern [7,8]. Most tumors also have central “comedo-like” necrosis which may mimic ductal carcinoma in situ of the breast, raising the differential diagnosis of a malignant adnexal carcinoma [4]. To confirm a diagnosis of PEA, immunohistochemical staining is key. Positive staining for S-100, carcinoembryonic antigen (CEA), and epithelial membrane antigen (EMA) support differentiation toward sweat gland secretory epithelium [9]. Although a strong S-100 reaction suggests eccrine gland differentiation, it is not always present and thus should not be relied upon solely for confirmation [10]. More dependable markers for indicating an eccrine origin of the tumor include alpha-smooth muscle actin (alpha-SMA), keratin 8, and keratin 14 [6].

It is crucial to differentiate between PEA and malignant adnexal carcinomas. As previously mentioned, PEA typically presents as a single, slow-growing, dome-shaped nodule located on the distal extremities, varying in colors. The characteristic features of PEA must be identified in order to prevent misdiagnosis of PEA as a carcinoma. Features suggestive of PEA include symmetry, branching tubular structures within the lumen, and a stroma distinctly separated from the dermis [8]. Adnexal carcinomas often present as smooth, flesh-colored or yellow, multicystic nodules that develop over several years. Adnexal carcinomas are often asymptomatic and most commonly located in the head and neck region, especially on the lips. They have rare metastatic potential; however, they can infiltrate locally [11]. Recurrence rates of adnexal carcinoma can be up to 47% in the first three years of diagnosis, which is why it is vital to have consistent follow-ups with patients; however, with Mohs surgery, recurrence can drop between 0% and 22% with a five-year follow up [11]. Trends have shown that an all-cause age survival of adnexal carcinoma has been 82% [12]. PEA can resemble BCC with similar tubules and 1-2 layers of cuboidal cells; however, they differ due to their cystic and alveolar areas as well as their local aggressiveness and lack of recurrence if excised with caution [6].

Treatment of PEA typically involves complete excision of the tumor with clear margins to prevent recurrence. Mohs micrographic surgery has also shown to be effective [6]. This patient did not have an excision after the initial biopsy, and his recurrence highlights the importance of recognizing this tumor and performing a prompt excision with appropriate margins. After excision with clear margins, recurrence and metastasis are rare; however, cases have been reported [13]. PEA is a rare benign eccrine tumor that can mimic adnexal carcinomas and necessitates careful pathologic examination and diagnosis to rule out more aggressive carcinomas.

## Conclusions

In conclusion, PEA is a rare, benign eccrine gland tumor that poses diagnostic challenges due to its resemblance to more aggressive adnexal carcinomas. Accurate histological diagnosis is essential to differentiate PEA from malignant tumors. While PEA typically follows a benign course, complete surgical excision with clear margins is crucial to prevent recurrence, as demonstrated in this case.
